# Effect of *Limosilactobacillus reuteri* DSM17938 to prevent antibiotic-associated diarrhea in children: prospective, multi-center, randomized, placebo-controlled clinical trial (PEARL Study)

**DOI:** 10.1007/s00431-025-06249-8

**Published:** 2025-06-09

**Authors:** Ener Cagri Dinleyici, Metehan Ozen, Sirin Guven, Nazan Dalgic, Adem Karbuz, Murat Sutcu, Ozden Turel, Fatma Nur Oz, Ulviye Kirli, Sevgi Yasar Durmus, Ahmet Sami Yazar, Zeynep Ebru Cakin, Yvan Vandenplas, Ates Kara

**Affiliations:** 1 Department of Pediatrics, Faculty of Medicine, Eskisehir Osmangazi University, Eskisehir, TR-26040 Türkiye; 2 Pediatric Infectious Disease Unit, Acıbadem University School of Medicine, Istanbul, Türkiye; 3 Department of Pediatrics, Prof. Dr. Ilhan Varank Training and Research Hospital, Istanbul, Türkiye; 4 Pediatric Infectious Disease Unit, Health Sciences University Sisli Hamidiye Etfal Training and Research Hospital, Istanbul, Türkiye; 5 Pediatric Infectious Disease Unit, Dr. Cemil Tascioglu City Hospital, Istanbul, Türkiye; 6 Pediatric Infectious Disease Unit, Istinye University Faculty of Medicine, Istanbul, Türkiye; 7 Pediatric Infectious Disease Unit, Istanbul Medeniyet University, Goztepe Education and Training Hospital, Prof Dr Suleyman Yalcin City Hospital, Istanbul, Türkiye; 8 Pediatric Infectious Disease Unit, Health Science University, Etlik City Hospital, Ankara, Türkiye; 9 Department of Pediatrics, Muğla Sıtkı Koçman University School of Medicine, Mugla, Türkiye; 10 Pediatric Infectious Disease Unit, Kayseri City Hospital, Kayseri, Türkiye; 11 Department of Pediatrics, VM Medical Park Hospital, Istanbul, Türkiye; 12 Faculty of Health Sciences, Mudanya University, Bursa, Türkiye; 13 Department of Pediatrics, Bagcilar Research and Training Hospital, Istanbul, Türkiye; 14 KidZ Health Castle, UZ Brussel, Vrije Universiteit Brussel, Brussels, Belgium; 15 Pediatric Infectious Disease Unit, Hacettepe University Faculty of Medicine, Ankara, Türkiye

**Keywords:** Antibiotic, Antibiotic associated diarrhea, Probiotic, Limosilactobacillus reuteri, L reuteri DSM 17938

## Abstract

Antibiotic-associated diarrhea (AAD) is one of the side effects that occur during and after antibiotic use. Some probiotics have strain-specific beneficial effects on AAD development when used in combination with antibiotics. The aim of this study was to evaluate the effect of *Limosilactobacillus reuteri* DSM 17938 on the prevention of AAD in children. This is a prospective, multicenter, randomized, double-blind, placebo-controlled clinical trial in Türkiye between 2017–2019, among outpatient children with acute otitis media (AOM) or acute rhinosinusitis (ARS). Group 1 (n = 330) received amoxicillin-clavulanate and *L. reuteri* DSM 17938 (2 × 10^8^ CFU) and Group 2 (n = 324) received amoxicillin-clavulanate and a placebo during the antibiotic treatment or continued for 21 days after antibiotic cessation. The primary end point of this study was the percentage of children with AAD in the first 14, 21, and 56 days of follow-up. Secondary endpoints are the percentage of children with AAD regarding the AOM vs ARS, amoxicillin-clavulanate dose, age groups, and the comparison between 14- and 21-days use of *L. reuteri*. The percentage of children with AAD was significantly lower in the *L. reuteri* group compared to the placebo group at 14 days (7.9% vs. 16.7%; RR: 0.47, 95%CI 0.30–0.7; *p* < 0.001); at 21 days (8.8% vs. 17.9%; RR: 0.49, 95%CI 0.32–0.74;*p* < 0.001); and at 56 days (9.1% vs. 19.6%; RR: 0.46, 95%CI 0.30–0.69;*p* < 0.001). The incidence of AAD was also significantly lower in the *L. reuteri* group at 14, 21 and 56 days among children aged between 6–24 months (*p* < 0.01, *p* < 0.01, *p* < 0.001) or children with AOM (p = 0.0001,*p* < 0.0001,*p* < 0.0001). When AAD was observed, the mean duration of diarrhea was longer in the placebo group(*p* < 0.05).

*Conclusions:* This first study with *L. reuteri* DSM 17938 in a large pediatric outpatient setting showed significant reduction of AAD during the first 14 days of antibiotic use and the 8-week follow-up period.

*Trial Registration*: NCT02765217 (First Submitted 02.05.2016) (https://clinicaltrials.gov/study/NCT02765217?term=NCT02765217&rank=1).

**Table:** 

**What is known:** • *Antibiotic associated diarrhea (AAD) is one of the common complications of antibiotic use in children and selected probiotics may have a strain-specific effect to prevent AAD* • *There are limited studies about the effects of Limosilactobacillus reuteri DSM 17938 on AAD, and no study available in pediatric outpatient setting.*	
**What is new:** • *Limosilactobacillus reuteri DSM 17938 significantly reduced the incidence of AAD in children at 14-, 21-, and 56- days follow-up.* • *The effect is mainly observed in children aged between 6 and 24 months or children with AOM.*

**Supplementary Information:**

The online version contains supplementary material available at 10.1007/s00431-025-06249-8.

## Introduction

Antibiotics are essential for treating severe acute bacterial infections; nevertheless, misuse can cause detrimental effects, including alterations to the composition and functioning of the human microbiota [1]. Furthermore, it is especially concerning since antibiotic usage throughout childhood is a risk factor for long-term effects such as obesity, allergies, inflammatory bowel disease, and behavioral disorders [1, 2]. Antibiotic associated diarrhea (AAD) is one of the common complications of antibiotic use, and is defined as diarrhea associated with antibiotic exposure, either while on antibiotics or for up to eight weeks after antibiotics have been discontinued [3]. The prevalence of pediatric AAD significantly varies based on two primary factors: the child’s age, particularly in those under two years, and the specific type of antibiotic administered, with a higher incidence associated with broad-spectrum antibiotics [3]. A prospective multi-center study involving pediatric patients who commenced an oral antibiotic regimen (predominantly aminopenicillins, 64%) in outpatient clinics in Türkiye from October 2016 to August 2018 revealed an incidence of AAD of 10.4% among 758 children aged 1 month to 12 years [4]. The clinical manifestation of AAD ranges from mild diarrhea to fulminant pseudomembranous colitis [5].

The prevention of AAD has conventionally depended on appropriate use of antibiotics, for instance, minimizing the use of broad-spectrum antibiotics whenever feasible [1, 3]. Preventive strategies to mitigate the risk of AAD also encompass the use of probiotics [1, 3, 6, 7]. Probiotics are defined as “live microorganisms, that when administered in adequate amounts, confer a health benefit on the host” [8]. Probiotics have been proposed to facilitate the restoration of a disrupted microbiota following antibiotic use; nevertheless, there is a lack of data establishing a causal relationship between these beneficial effects and microbiota protection or recovery [1]. Selected probiotics may have a moderate effect to prevent AAD in children, adults, and elderly [6]. A meta-analysis of several probiotics for the prevention of pediatric AAD identified two probiotics that were particularly effective: *Saccharomyces boulardii* CNCM I-745 and *Lacticaseibacillus rhamnosus* GG (LGG) [9]. The recent position paper of the European Society for Paediatric Gastroenterology, Hepatology and Nutrition (ESPGHAN) indicates that if the use of probiotics for preventing AAD is considered, healthcare professionals may recommend high doses of *Saccharomyces boulardii* or *LGG* started simultaneously with antibiotic treatment to prevent AAD in both outpatients and hospitalized children [7]. Nonetheless, consistent with the position that probiotic effects are strain-specific, the efficacy and safety of each probiotic strain should be determined independently [7]. The strains and doses of probiotics, characteristics of the host, and the definition of clinical end point might affect the effects of probiotics on AAD [3, 7, 10].

*Limosilactobacillus (L.) reuteri* DSM 17938 (formerly known as *Lactobacillus reuteri*) was first reported in the early 1980 s and is a natural member of the gut microbiota [11, 12]. Preclinical research has shown that this probiotic can ameliorate dysbiosis [12]. Previous clinical trials showed that *L. reuteri* DSM 17938 has some beneficial effects on functional gastrointestinal disorders, such as colic and regurgitation in infants, functional abdominal pain, and functional constipation in children and adolescents [11, 12]. *L. reuteri* DSM 17938 can decrease the duration of acute diarrhea and hospitalization for acute infectious diarrhea in children [13]. Because of its ability to survive in the gastric environment, it has been tested in *Helicobacter pylori* infection, showing a significant decrease in antibiotic-associated side effects and a tendency to increase the eradication rate [14]. Finally, all these studies have shown the excellent safety profile of this strain [11–14]. There are limited studies about the effects of *L. reuteri* on AAD. There is no previous study in a pediatric outpatient setting. We aimed to assess the effectiveness of *L. reuteri* DSM 17938 administration for the prevention of diarrhea and AAD in an outpatient population of children.

## Methods

**Study Design:** The PEARL study is an investigator-initiated, prospective, multi-center, double-blind, placebo-controlled, randomized clinical trial that was performed in Türkiye between 2017 and 2019. The study was prospectively registered in the ClinicalTrials.gov database (NCT02765217). The PEARL study was approved by the Sisli Hamidiye Etfal Training and Research Hospital Clinical Research Local Ethical Committee (15 March 2016, Decision Number 1124). The study was conducted according to current Consolidated Standards of Reporting Trials (CONSORT) reporting guidelines.

**Participants:** We have enrolled children with acute otitis media (AOM) or acute rhinosinusitis (ARS) aged between six months and 10 years. The inclusion criteria were children receiving amoxicillin-clavulanate due to AOM or ARS. Exclusion criteria were receiving antibiotic and/or probiotic within eight weeks prior to the study; chronic gastrointestinal system disorders (chronic diarrhea, coeliac disease, inflammatory bowel disease); the presence of congenital anomalies; presence of immune deficiency; and receiving chronic medications due to any cause.

**Randomization and Masking:** The design of this study involves a randomized, double-blind, placebo-controlled trial with a 1:1:1:1 allocation. Randomization was done centrally in blocks of eight by probiotic (10–14 days), probiotic (21 days), placebo (10–14 days), or placebo (21 days). A computer random-sequence generator was used by the principal investigator. Participants will receive consecutive randomization numbers at enrolment. The participants, caregivers, and all investigators were blinded until the data analysis was performed.

**Intervention:** Written informed consent was obtained by the physicians involved in the study. All children were treated with amoxicillin-clavulanate (oral suspension, 50 or 90 mg/kg/day, twice daily, for 10 to 14 days). Enrolled patients also received either *L. reuteri* DSM 17938 at a dose of 10^8^ CFU or placebo, orally, twice daily, in drops (2 × 5 drops), during the entire period of antibiotic treatment (10- 14 days) or continued for 21 days.

- **Group 1** received amoxicillin-clavulanate and ***L. reuteri* DSM 17938** twice daily, 10^8^ CFU, during the antibiotic treatment (**10–14 days; Group 1a**) or continued (**for 21 days; Group 1b**) after cessation of the antibiotic treatment.
- **Group 2** received amoxicillin-clavulanate and **placebo** during the antibiotic treatment (**10–14 days; Group 2a**) or continued (**for 21 days; Group 2b**) after cessation of antibiotic treatment.

The study products (*L. reuteri* DSM 17938 and placebo) were manufactured and supplied by BioGaia (Lund, Sweden), free of charge. The placebo drops were identical to the probiotic product but without *L. reuteri* DSM 17938. The packaging of the probiotic and placebo was identical, exhibiting the same appearance, taste, and smell.

**Outcomes:** Throughout the study period, parents or caregivers recorded the number and consistency of stools in a standard stool diary for 56 days. The frequency and consistency of the stools were recorded daily according to the Bristol stool scale; a score equal to or above Type 5 was defined as diarrhea. When diarrhea occurred, we asked as a part of study protocol, the participants’caregivers to provide stool samples for testing for the infectious pathogen. If we detected viruses or bacteria as the cause of diarrhea, we excluded the patient from the study. AAD was defined as ≥ 3 loose or watery stools per day for a minimum of 48 h during the antibiotic treatment and up to eight weeks antibiotic use. We will follow up all study participants for the duration of the antibiotic treatment and then for 56 days after the intervention.

The primary endpoint of this study was the comparison of the percentage of children with AAD during 14, 21 and 56 days after the start of the antibiotic treatment between the *L. reuteri DSM 17938* and placebo group. The secondary end-points were (1) subgroup analysis including comparison of percentage of children with AAD in first 14, 21 and 56 days follow-up between the *L. reuteri DSM 17938* and placebo groups according to the age groups (6–24 months, 25–59 months and 60–120 months); (2) subgroup analysis including comparison of percentage of children with AAD in first 14, 21 and 56 days follow-up between *L. reuteri DSM 17938* and placebo group in children with AOM or children with ARS; (3) subgroup analysis including comparison of percentage of children with AAD in first 14, 21 and 56 days follow-up between *L. reuteri DSM 17938* and placebo group in children regarding to amoxicillin-clavulanate dose (50 mg/kg/day or 90 mg/kg/day). We also performed a comparison between the efficacy of *L. reuteri DSM 17938* for 10–14 days and 21 days in the probiotic group. We also evaluated the disease course of children with AAD: duration of diarrhea, presence of dehydration, requiring medical care, and requiring hospitalization, if available.

**Statistical Analysis:** Based on the pooled risks of AAD determined from the previous studies about AAD, we expected that the incidence of AAD would be 20% among children receiving placebo. We calculated that 720 people (360 probiotic and 360 placebo, with two subgroups in each) were needed to find a 10% difference between the groups at a 5% significance level, with 90% power, and a 20% chance of dropping out. We only performed per-protocol analysis because AAD incidence during the 56-day follow-up is one of the primary outcomes. Data analyses were conducted using the JASP statistical program. For continuous outcomes, differences in means or medians (depending on the distribution of the data) have been evaluated. The χ^2^ test or Fisher’s exact test will be used, as appropriate, to compare percentages. For dichotomous outcomes, the relative risk (RR) with a 95% CI and number needed to treat (NNT) will be calculated. The difference between study groups will be considered significant when the p-value is < 0.05, when the 95% CI for RR does not include 1.0, or when the 95% CI for mean difference does not include 0. All statistical tests will be two-tailed and performed at the 5% level of significance.

## Results

Totally, 720 participants have been enrolled, and the drop-out rate was 9.2% (n = 66). The flowchart of the study and the reasons for exclusion of infants are given in Supplementary Fig. 1. During the 56 days of the study, diarrhea occurred in 122 children (42 in the *L. reuteri* DSM 17938 group and 82 in the placebo group). Among nine children (four in the *L. reuteri* DSM 17938 group and five in the placebo group) who developed diarrhea, stools tested positive for acute infectious diarrhea and were excluded from the analysis. Eight children in the *L. reuteri* DSM 17938 group and 11 children in the placebo group did not provide a stool sample for further tests. All these patients with diarrhea (n:28) were not defined as AAD cases for the per-protocol analysis. Per-protocol analysis was carried out in 654 children who have been followed up during the entire study period.

The study group consists of 330 children (Group 1; 167 boys, 163 girls; mean age, 43.9 ± 32.6 months) in the *L. reuteri DSM 17938* group and 324 children (Group 2; 156 boys, 168 girls; mean age, 43.5 ± 27.2 months) in the placebo group. Characteristics of the *L. reuteri DSM 17938* and placebo groups were summarized in Table 1. 65.4% of children in the *L. reuteri DSM 17938* group had a diagnosis of AOM, and 60.2% of children in the placebo group. In the *L. reuteri DSM 17938* group, 71.5% (n = 236) of children received amoxicillin-clavulanate at the dose of 50 mg/kg/day and 71% (n = 230) in the placebo group. Gender, age distribution, presence of acute otitis media or acute rhinosinusitis, and daily dose of amoxicillin-clavulanate (50 mg/kg/day vs. 90 mg/kg/day) were similar between the *L. reuteri DSM 17938* group and the placebo group (p > 0.05 for all) (Table 1). In the *L. reuteri DSM 17938* group, 168 children received *L. reuteri* DSM 17938 twice daily for 14 days (Group 1a), and 162 children received for 21 days (Group 1b). In the placebo group, 165 children received placebo for 14 days (Group 2a), and 159 children received it for 21 days (Group 2b). There is no difference in the percentage of children, gender, age, infection, or amoxicillin-clavulanate dose between the probiotic and placebo subgroups for 14 days (Group 1a vs. 2a) and 21 days (Group 1b vs. 2b) (p > 0.05 for all) (Table 1).

**Table 1:** Demographical characteristics, infectious disease and antibiotic dose of study groups

	*L. reuteri DSM 17938 group* *Group 1 (n* = *330)*			*Placebo group* *Group 2 (n* = *324)*		
Characteristics	Group 1a 14 days (*n* = 168)	Group 1b 21 days (*n* = 162)	Total (*n* = 330)	Group 2a 14 days (*n* = 165)	Group 2b 21 days (*n* = 159)	Total (*n* = 324)
*Gender (M/F)*	87/81	81/81	167/163	72/93	83/76	155/169
Age (months), mean (SD)	42.8 ± 33.0	45.0 ± 32.3	43.9 ± 32.6	46.5 ± 28.0	40.4 ± 26.0	43.5 ± 27.2
Age distribution						
≥ 6 to 24 months, *n* (%)	78 (45.4%)	69 (42.6%)	147 (44.6%)	59 (35.8%)	66 (41.5%)	125 (38.6%)
≥ 25 to 60 months, *n* (%)	35 (20.8%)	39 (24.1%)	74 (22.4%)	47 (28.4%)	52 (32.7%)	99 (30.6%)
≥ 61 months to 10 years, *n* (%)	55 (32.8%)	54 (33.3%)	109 (33.0%)	59 (35.8%)	41 (25.8%)	100 (30.8%)
Infection						
Acute otitis media	113 (67.2%)	106 (65.4%)	219 (66.4%)	90 (54.5%)	105 (66.0%)	195 (60.2%)
Acute rhinosinusitis	55 (32.8%)	56 (34.6%)	111 (33.6%)	75 (45.5%)	54 (34.0%)	129 (39.8%)
Amoxicillin-clavulanate						
50 mg/kg/day BID *n* (%)	121 (72.0%)	115 (71.0%)	236 (71.5%)	125 (75.8%)	105 (66.0%)	230 (71.0%)
80–90 mg/kg/day BID *n* (%)	47 (28.0%)	47 (29.0%)	94 (28.5%)	40 (24.2%)	54 (34.0%)	94 (29.0%)

**Primary outcome:** The percentage of children with AAD was significantly lower in the *L. reuteri* DSM 17938 group compared to the placebo group: 14 days: 26/330 (7.9%) vs. 54/324 (16.7%), RR: 0.47 (95% CI 0.30–0.7; *p* < 0.001; NNT 11); 21 days: 29/330 (8.8%) vs. 58/324 (17.9%), RR: 0.49 (95% CI 0.32–0.74; *p* < 0.001; NNT 11); 56 days: 30/330 (9.1) vs. 64/324 (19.6%), RR: 0.46 (95% CI 0.30–0.69; *p* < 0.001; NNT 9) (Table 2).

**Table 2:** Presence of AAD on the 14, 21 and 56 day of study between the *L. reuteri* DSM 17938 group and the placebo group

AAD	*L. reuteri* DSM 17938 group Group 1 (*n* = 330)	Placebo group Group 2 (*n* = 324)	RR (95% CI) *p* value NNT
14 days	26/330 (7.9%)	54/324 (16.7%)	**0.47 (95%CI 0.30–0.73)** ***p*** **< 0.001** **NNT 11**
21 days	29/330 (8.8%)	58/324 (17.9%)	**0.49 (95%CI 0.32–0.74)** ***p*** **< 0.001** **NNT 11**
56 days	30/330 (9.1%)	64/324 (19.6%)	**0.46 (95%CI 0.30–0.69)** ***p*** **< 0.001** **NNT: 9**

*L. reuteri: Limosilactobacillus reuteri*, *AAD* Antibiotic associated diarrhea, *RR* relative risk, *CI* Confidence interval, *NNT* number needed to treat

**Secondary outcomes: **When we compared 14 days of use of the *L. reuteri* DSM 17938 group (1a) with 14 days of use of the placebo (2a), the incidence of AAD was significantly lower at 14 days (7.7% vs. 15.2%; *p* < 0.05, NNT 12). While the incidence of AAD was lower at 21 and 56 days in the *L. reuteri* DSM 17938 group, there is no statistical significance (p > 0.05 for both). When we compared 21 days of use of *L. reuteri* DSM 17938 (2a) with 21 days of placebo (2b); the incidence of AAD was significantly lower at 14 days (8.0% vs. 18.9%; *p* < 0.001, NNT 7), 21 days (8.6% vs. 20.1%; *p* < 0.01, NNT 9), and 56 days (8.6% vs. 23.9%, *p* < 0.001, NNT 7) in the *L. reuteri* DSM 17938 group compared to the placebo group (Table 3).

**Table 3:** Comparison of incidence of AAD between 14 days use of *L. reuteri* DSM 17938 group (1a) and placebo (2a) or 21 days use of *L. reuteri* DSM 17938 (1b) group and placebo (2b)

AAD 14 days use of L. reuteri or placebo	*L. reuteri* DSM 17938 group Group 1a (*n* = 168)	Placebo group Group 2a (*n* = 165)	RR (95% CI) *p* value NNT
14 days of study	13/168 (7.7%)	25/165 (15.2%)	**0.51 (95%CI 0.27–0.96)** ***p*** **= 0.038** NNT 13
21 days of study	15/168 (8.9%)	26/165 (15.8%)	0.56 (95%CI 0.31–1.03) *p* = 0.0627
56 days of study	16/168 (9.5%)	26/165 (15.8%)	0.60 (95%CI 0.33–1.08) *p* = 0.0914
AAD 21 days use of L. reuteri or placebo	***L. reuteri* DSM 17938 group** **Group 1b (*n*** **= 162)**	**Placebo group** **Group 2b (*n*** **= 159)**	**RR (95% CI)** ***p*** **value** **NNT**
14 days of study	13/162 (8.0%)	30/159 (18.9%)	**0.34 (95%CI 0.19–0.62)** ***p*** **< 0.001** **NNT: 7**
21 days of study	14/162 (8.6%)	32/159 (20.1%)	**0.42 (95%CI 0.23–0.77)** ***p*** **< 0.01** **NNT: 9**
56 days of study	14/162 (8.6%)	38/159 (23.9%)	**0.36 (95%CI 0.20–0.64)** ***p*** **< 0.001** **NNT: 7**

*L. reuteri: Limosilactobacillus reuteri*, *AAD* Antibiotic associated diarrhea, *RR* relative risk, *CI* Confidence interval, *NNT* number needed to treat

We subdivided the study group according to age as follows: ≥ 6 to 24 months, ≥ 25 to 60 months, and ≥ 61 months to 10 years. Among children aged between 6 and 24 months, the incidence of AAD was significantly lower in the *L. reuteri* DSM 17938 group compared to the placebo group at 14 days (6.8% vs. 20.8%; *p* < 0.01, NNT 7), at 21 days (8.8% vs. 22.4%; *p* < 0.01, NNT 7), and at 56 days (9.5% vs. 25.6%; *p* < 0.001, NNT 6). While the incidence of AAD is lower in the *L. reuteri* DSM 17938 group, there is no statistical difference observed in the ≥ 25 to 60 months and ≥ 61 months to 10 years groups at 14, 21, and 56 days of study (p > 0.05 for all) (Table 4).

**Table 4:** Comparison of AAD incidence between the *L. reuteri* DSM 17938 group and the placebo regarding age groups

≥ 6 to 24 months	*L. reuteri* DSM 17938 group Group 1 (*n* = 147)	Placebo group Group 2 (*n* = 125)	RR (95% CI) *p* value NNT
14 days of study	10/147 (6.8%)	26/125 (20.8%)	**0.32 (95%CI 0.16–0.65)** ***p*** **< 0.01** **NNT 7**
21 days of study	13/147 (8.8%)	28/125 (22.4%)	**0.39 (95%CI 0.21–0.72)** ***p*** **< 0.01** **NNT 7**
56 days of study	14/168 (9.5%)	32/125 (25.6%)	**0.37 (95%CI 0.20–0.66)** ***p*** **< 0.001** **NNT 6**
≥ 25 to 60 months	***L. reuteri* DSM 17938 group** **Group 1 (*n*** **= 74)**	**Placebo group** **Group 2 (*n*** **= 99)**	**RR (95% CI)** ***p*** **value** **NNT**
14 days of study	9/74 (12.2%)	20/99 (20.2%)	0.60 (95%CI 0.29–1.24) *p* = 0.1712
21 days of study	9/74 (12.2%)	22/99 (22.2%)	0.54 (95%CI 0.26–1.11) *p* = 0.0983
56 days of study	9/74 (12.2%)	22/99 (22.2%)	0.54 (95%CI 0.26–1.11) *p* = 0.0983
≥ 61 months to 10 years	***L. reuteri* DSM 17938 group** **Group 1b (*n*** **= 109)**	**Placebo group** **Group 2b (*n*** **= 100)**	**RR (95% CI)** ***p*** **value** **NNT**
14 days of study	7/109 (6.4%)	8/100 (8.0%)	0.80 (95%CI 0.30–2.13) *p* = 0.6595
21 days of study	7/109 (6.4%)	8/100 (8.0%)	0.80 (95%CI 0.30–2.13) *p* = 0.6595
56 days of study	7/109 (6.4%)	8/100 (8.0%)	0.80 (95%CI 0.30–2.13) *p* = 0.6595

*L. reuteri: Limosilactobacillus reuteri*, *AAD* Antibiotic associated diarrhea, *RR* relative risk, *CI* Confidence interval, *NNT* number needed to treat

219 children in the *L. reuteri* DSM 17938 group and 195 children in the placebo group were diagnosed with AOM. Among children diagnosed with AOM, the incidence of AAD was significantly lower in the *L. reuteri* DSM 17938 group compared to the placebo group at 14 days of study (8.7% vs. 23.0%; p = 0.0001, NNT 7), 21 days of study (10.0% vs. 24.6%; *p* < 0.0001, NNT 7), and 56 days of study (10.5% vs. 27.7%; *p* < 0.0001, NNT 6). In the study group, 111 children in the *L. reuteri* DSM 17938 group and 129 children in the placebo group were diagnosed with ARS. Among children diagnosed with ARS, there is no difference for AAD between the *L. reuteri* DSM 17938 group and the placebo group at the 14, 21, and 56 days of the study (p > 0.05) (Table 5).

**Table 5:** Comparison of AAD incidence between the *L. reuteri* DSM 17938 group and the placebo in children with acute otitis media or acute rhinosinusitis and in children receiving amoxicillin-clavulanate at the dose of 50 mg/kg/day or 90 mg/kg/day

Acute otitis media	*L. reuteri* DSM 17938 group Group 1 (*n* = 219)	Placebo group Group 2 (*n* = 195)	RR (95% CI) *p* value, NNT
14 days of study	19/219 (8.7%)	45/195 (23.0%)	**0.37 (95%CI 0.22–0.62)** ***p*** **= 0.0001, NNT 7**
21 days of study	22/219 (10.0%)	48/195 (24.6%)	**0.40 (95%CI 0.25–0.65)** ***p*** **< 0.001, NNT 7**
56 days of study	23/219 (10.5%)	54/195 (27.7%)	**0.37 (95%CI 0.24–0.59)** ***p*** **< 0.0001, NNT 6**
Acute Rhinosinusitis	***L. reuteri* DSM 17938 group** **Group 1 (*n*** **= 111)**	**Placebo group** **Group 2 (*n*** **= 129)**	**RR (95% CI)** ***p*** **value, NNT**
14 days of study	7/111 (6.3%)	9/129 (7.0%)	0.90 (95%CI 0.34–2.34) *p* = 0.8357
21 days of study	7/111 (6.3%)	10/129 (7.8%)	0.81 (95%CI 0.32–2.06) *p* = 0.6642
56 days of study	7/111 (6.3%)	10/129 (7.8%)	0.81 (95%CI 0.32–2.06) *p* = 0.6642
Amoxicillin-clavulanate 50 mg/kg/day	***L. reuteri* DSM 17938 group** **Group 1 (*n*** **= 236)**	**Placebo group** **Group 2 (*n*** **= 230)**	**RR (95% CI)** ***p*** **value, NNT**
14 days of study	18/236 (7.6%)	37/230 (16.1%)	**0.47 (95%CI 0.27–0.80)** ***p*** **< 0.01, NNT 12**
21 days of study	19/236 (8.0%)	39/230 (17.0%)	**0.47 (95%CI 0.28–0.79)** ***p*** **< 0.01, NNT 11**
56 days of study	20/236 (8.5%)	41/230 (17.8%)	**0.47 (95%CI 0.28–0.78)** ***p*** **< 0.01, NNT 11**
Amoxicillin-clavulanate 90 mg/kg/day	***L. reuteri* DSM 17938 group** **Group 1 (*n*** **= 94)**	**Placebo group** **Group 2 (*n*** **= 94)**	**RR (95% CI)** ***p*** **value, NNT**
14 days of study	8/94 (8.5%)	17/94 (18.1%)	0.47 (95%CI 0.21–1.03) *p* = 0.0615
21 days of study	10/94 (10.6%)	19/94 (20.2%)	0.52 (95%CI 0.25–1.07) *p* = 0.0766
56 days of study	10/94 (10.6%)	23/94 (24.5%)	**0.43 (95%CI 0.21–0.86)** ***p*** **< 0.05, NNT 7**

*L. reuteri: Limosilactobacillus reuteri*, *AAD* Antibiotic associated diarrhea, *RR* relative risk, *CI* Confidence interval, *NNT* number needed to treat

In the *L. reuteri* DSM 17938 group, for AAD, there is no difference for AAD between the 10–14 days group (1a) and the 21 days group (1b) (14 days of study: 13/168 (7.7%) vs. 13/162 (8.0%); 21 days of study: 15/168 (8.9%) vs. 14/162 (8.6%); 56 days of study: 16/168 (9.5%) vs. 14/162 (8.6%); p > 0.05 for all).

There is also no difference between 14 and 21 days of placebo at 14, 21 and 56 days (p > 0.05 for all). 86.6% of AAD in the *L. reuteri* DSM 17938 group occurred in the first 14 days, compared to 84.3% of AAD in the placebo group. When AAD was observed; the mean duration of diarrhea was longer in the placebo group than in the *L. reuteri* group (3.2 ± 0.93 days vs. 2.8 ± 0.99 days, *p* < 0.05). We did not observe dehydration, emergency care unit admission, or hospitalization in the *L. reuteri* DSM 17938 group or in the placebo group*. L. reuteri DSM 17938* is well tolerated, there are no dropouts related to probiotic use.

## Discussion

In this large, placebo-controlled, double-blind, randomized clinical trial, *L. reuteri* DSM 17938 reduced the incidence of AAD on 14, 21, and 56 days of follow-up compared to the placebo group. This is the largest clinical trial showing the effect of *reuteri* DSM 17938 for the prevention of AAD in children and the first study in an outpatient setting. The NNT is 11 at the 14 and 21 days of follow-up and nine at the 56 days of follow-up.

There are two previous studies about the effects of *L. reuteri* DSM 17938 on the prevention of AAD in children; both were performed on hospitalized children [15, 16]. A study by Georgieva et al. [15] enrolled 97 hospitalized children in Bulgaria and found that there was no significant difference in the risk of AAD between the *L. reuteri* DSM 17938 and placebo groups. However, the overall frequency of diarrhea was very low, with one case in each study group [15]. Kolodzej and Szajewska [16] also assessed the effects of *L. reuteri* DSM 17938 (a dose of 10^8^ CFU orally, twice daily, for the entire duration of antibiotic treatment) on the prevention of diarrhea and AAD in 247 hospitalized children in a double-blind, randomized, placebo-controlled trial. The occurrences of diarrhea and AAD were similar in the *L. reuteri* DSM 17938 and placebo groups, regardless of the definition used, including the definition of AAD as three or more loose or watery stools per day for a minimum of 48 h, like our study [16]. There are differences between the present study and these two studies for the follow-up period, definition of AAD and stool consistency, and dose of probiotics. The main difference is that both studies have been performed in hospitalized children and children receiving different groups of oral/parenteral antibiotics due to different causes of infection [15, 16]. There are also previous studies about the effects of *L. reuteri* DSM 17938 in adult patients for *Helicobacter pylori* eradication, and they showed that *L. reuteri* DSM 17938 reduced gastrointestinal symptoms, including diarrhea associated with the use of antibiotics for the treatment of HP infection, with an increase in eradication rate [12, 14]. Our study is the first study in a pediatric outpatient setting, and *L. reuteri* DSM 17938 (2 × 10^8^ CFU, daily) significantly reduced the AAD incidence at three time points of the study in children receiving amoxicillin-clavulanate due to AOM or ARS. Concomitant use of *L. reuteri* DSM 17938 with antibiotics is not a concern of the present study, because *L. reuteri* DSM 17938 is not sensitive to amoxicillin-clavulanate [17].

In comparison to adults, children generally exhibit symptoms rapidly during antibiotic therapy. Nonetheless, their recovery is expedited, they experience fewer problems, and their disease duration is shorter in comparison to adults [3]. The incubation period for AAD (defined as the interval between the commencement of antibiotic therapy and the emergence of diarrhea) predominantly occurs during antibiotic treatment [3, 4]. The mean incubation period for pediatric antibiotic-associated diarrhea (AAD) ranges from 2 to 6 days, with AAD generally manifesting during antibiotic treatment in 85% to 92% of cases; only 8% to 15% report delayed-onset AAD following drug cessation [3]. In our study, we observed the majority of the diarrhea during the first 7 days of antibiotic treatment. Clinical remission is fast in our study group, and the duration of diarrhea is shorter in children receiving *L. reuteri* DSM 17938 than placebo (3.2 ± 0.93 days vs. 2.8 ± 0.99 days). The previously reported mean duration for pediatric AAD is 3–9 days [3]. We previously performed two multicenter randomized clinical trials in children with acute infectious diarrhea that showed that *L. reuteri* DSM 17938 reduced the duration of diarrhea in outpatient or hospitalized children [13]. This might be related to the beneficial effects of probiotics on intestinal microbiota composition on diarrhea. Like previous studies with *L. reuteri* DSM 17938 for different clinical conditions, *L. reuteri* DSM 17938 is well-tolerated in children, and no dropouts have been reported due to probiotic use in our study cohort.

The beneficial effect of *L. reuteri* DSM 17938 is mainly observed in children aged between 6 and 24 months or children with AOM. The incidence of pediatric AAD varies largely due to two main factors: the age of the child (below two years old) and the type of antibiotic to which the child is exposed [3]. In the present study, *L. reuteri* DSM 17938 reduced the incidence of AAD in infants aged between 6 and 24 months of age by approximately 61–68% during the 56-day follow up and NNT was 6–7 in this age group. More than two-thirds of children receive antibiotics before reaching the age of two years, with exposure to an average of almost three antibiotics in the first year of life [18]. These detrimental consequences are likely attributable to alterations in the gut microbiota, which experiences substantial maturation throughout the initial two to three years of life. The initial years of life are pivotal for the establishment and maintenance of the gut microbiota, aligning with significant milestones in immune system development, metabolism, and neurodevelopment [18, 19]. Consequently, any disturbances of the microbiota during this critical period may have long-lasting and far-reaching consequences.

We also observed a reduction of AAD in children with AOM; no effects have been observed in children with ARS. In our study, in the placebo group, the incidence of AAD is higher (23–27.7%) in children with AOM compared to the ARS cases (7–7.8%). The difference in the effect of *L. reuteri* DSM 17938 in children with AOM and ARS might not only be related to the probiotic but also to the infectious disease/course itself. In Türkiye, regarding the previous study about the incidence of AAD in children, the most common antibiotic groups prescribed were aminopenicillins (64%), and AAD incidence is higher in patients treated with the high dose compared to the usual dose [4]. In our study, *L. reuteri* DSM 17938 significantly reduced AAD in children receiving the usual dose of 50 mg/kg/day, on the 14 days, 21 days and 56 days of the study. In higher doses of amoxicillin-clavulanate, clinical benefits have been observed only at 56 days. For this reason, selection of patients requiring high-dose antibiotics is crucial. These results highlighted the importance of host and treatment-related factors when evaluating the probiotics on clinical conditions. Infectious disease type, antibiotic class, oral or parenteral route, and host age might affect the effectiveness of the probiotics. While antibiotics are commonly used drugs among children for the prevention of AAD, clinicians prefer to select appropriate cases for probiotic use.

The exact mechanism of *L. reuteri* DSM 17938 for prevention of AAD is not clear. *L. reuteri* DSM 17938 can colonize primarily the gastrointestinal tract, adhere to the intestinal epithelium, and produce antimicrobial molecules, strengthening the gut barrier and decreasing the microbial translocation from the intestinal lumen to the tissue [12]. *L. reuteri* DSM 17938 improves AAD and infectious diarrhea, inhibiting the growth of rotavirus in infants and the growth of bacteria in vitro and in vivo studies [11, 12]. *L. reuteri* has also produced mucin, reuterin, and antioxidant substances and has some potential beneficial effects on gut microbiota [12]. Previous studies have shown that probiotics may exert positive effects on the composition of intestinal microbiota in patients with acute infectious diarrhea or AAD [1, 20, 21]. The impact of antibiotics on the composition and function of the human microbiota is extensively studied. However, there has been little consideration of the strength of evidence supporting the hypothesis that probiotics can help restore the antibiotic-disrupted microbiota. Probiotics have been suggested to facilitate the restoration of a disrupted microbiome following antibiotic administration. Any serious consideration of this hypothesis must consider multiple factors that affect such outcomes, including the strains and doses of probiotics being administered, characteristics of the host (such as host genotype and microbiota), methodologies used to assess microbiota recovery, and measurement of microbiological as well as clinical end points, among others [1]. The wide range of benefits that probiotics offer during antibiotic treatment, as well as the ways that these clinical benefits work, are still not fully understood. The degree to which probiotic-induced microbiota protection or restoration influences clinical outcomes remains speculative. This study did not include microbiome analysis. To prevent AAD, in addition to restoring microbiota using probiotics, further well-conducted clinical trials are necessary.

### Strengths and Limitations

The strengths of this trial are the first study in an outpatient setting, placebo-controlled and representative study population for the pediatric age group. To our knowledge, this is one of the largest clinical trials investigating the effect of a probiotic on the incidence of AAD in children. We selected only outpatient children, a single antibiotic, and the standard two doses. While a 56-day follow-up period was required, loss to follow-up was lower than expected. The overall occurrence of AAD in the placebo group ranged from 16.7% to 19.6%; it was generally in line with our initial assumptions, and the study was not underpowered.

This study also has some limitations. First, postponement of study due to lockdown restrictions due to the COVID-19 pandemic led to not performing the second part of the PEARL study. The second part plans to evaluate the non-concomitant use of probiotics and antibiotics (six hours distance between the use); however, this postponement is not related to this present part of the study. We reached the target enrollment number for this concomitant use study. We used the Bristol stool score for the definition of diarrhea, and this score has some limitations in infancy. We also did not perform microbiota analysis for these children.

## Conclusions

Results of the PEARL study showed that *L. reuteri* DSM 17938 did reduce the incidence of AAD in children during the 14, 21, and 56 days of follow-up. The effect is mainly observed in children receiving 21 days of probiotics or children aged between 6 and 24 months or children with AOM. The effects of probiotics in preventing AAD are strain-specific and may also vary according to the antibiotic used. In our study, we demonstrated the positive effects of *L. reuteri* DSM 17938 in combination with amoxicillin-clavulanic acid. Since it would not be possible to generalize these findings to all antibiotics, further studies are needed to assess its use with other antibiotics. Antibiotics are essential for treating serious bacterial infections; however, their abuse can cause harm, including detrimental effects on microbiota composition. The prevention of AAD has conventionally depended on the appropriate usage of antibiotics, such as minimizing the use of broad-spectrum antibiotics whenever feasible. Few probiotic strains have been shown to effectively prevent pediatric AAD, and *L. reuteri* DSM 17938 could be used as an additional option.

## Supplementary Information

Below is the link to the electronic supplementary material.

ESM 1 (DOCX 583 KB)

## Data Availability

The data collected during this study are available from the corresponding author upon reasonable request (after publication).
