# Supplementary figures and images for: Effect of Limosilactobacillus reuteri DSM17938 to prevent antibiotic-associated diarrhea in children: prospective, multi-center, randomized, placebo-controlled clinical trial (PEARL Study)
Source: Eur J Pediatr. 2025 Jun 9;184(7):408. doi: 10.1007/s00431-025-06249-8 (PMC12149013; doi:10.1007/s00431-025-06249-8)

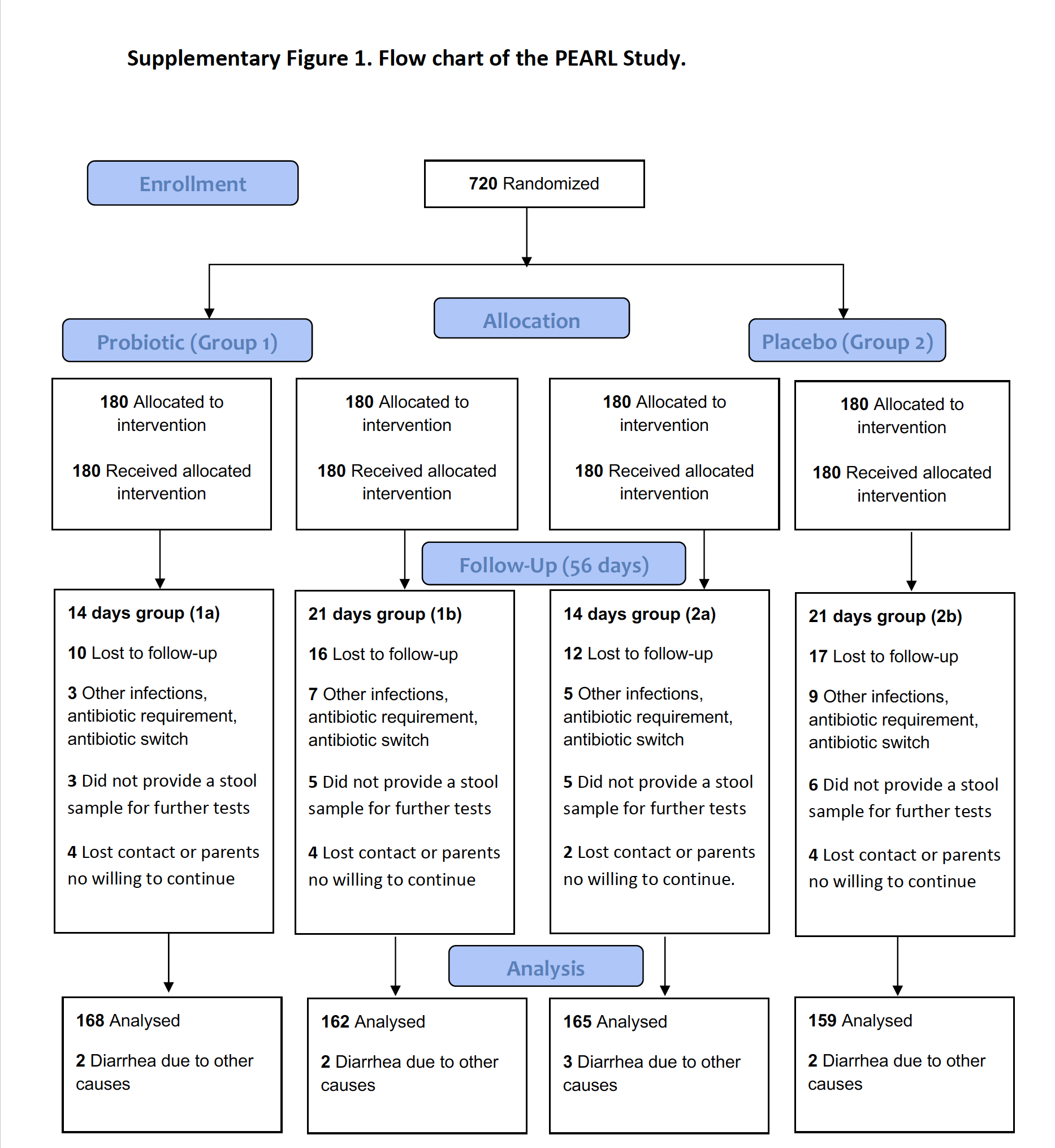

Supplement: Supplementary file 1 — (DOCX 583 KB) [file 431_2025_6249_MOESM1_ESM.docx]
